# Changes in health behaviors and obesity of Korean adolescents before and during the COVID-19 pandemic: a special report using the Korea Youth Risk Behavior Survey

**DOI:** 10.4178/epih.e2023018

**Published:** 2023-02-14

**Authors:** Chang-Mo Oh, Yangha Kim, Jieun Yang, Sunhye Choi, Kyungwon Oh

**Affiliations:** 1 Department of Preventive Medicine, Kyung Hee University School of Medicine, Seoul, Korea; 2 Division of Health and Nutrition Survey and Analysis, Bureau of Chronic Disease Prevention and Control, Korea Disease Control and Prevention Agency, Cheongju, Korea

**Keywords:** Health surveys, Adolescent, Health behavior, Obesity, COVID-19

## Abstract

**OBJECTIVES:**

This study aimed to investigate changes in health behaviors, including cigarette smoking, alcohol drinking, physical activity, dietary behaviors, and obesity, before and during the coronavirus disease 2019 (COVID-19) pandemic using the Korea Youth Risk Behavior Survey (KYRBS) database.

**METHODS:**

KYRBS data from 2015 to 2021 were used in this study. Differences in health behaviors between before (pre-pandemic period: 2018-2019) and during (pandemic period: 2020-2021) the pandemic were examined. Differences were compared using linear regression and the chi-square test considering the complex survey design after adjusting for grade level.

**RESULTS:**

The prevalence of current cigarette smoking and current alcohol drinking significantly decreased in both male and female students during the pandemic compared to the pre-pandemic period. However, the prevalence of obesity significantly increased in both male and female students during the same period. When examining physical activity and dietary behaviors closely related to obesity, fast food consumption increased and fruit consumption decreased during the pandemic in both male and female students, whereas no significant changes in physical activity were observed in either male or female students.

**CONCLUSIONS:**

The deterioration of adolescent dietary behaviors and an increase in the prevalence of obesity can increase the future disease burden, and concerted efforts at the individual and national levels are needed to reduce obesity and promote healthy dietary behaviors.

## GRAPHICAL ABSTRACT

[Fig f4-epih-45-e2023018]

## INTRODUCTION

Lifestyle and health-related behaviors during adolescence not only translate into the health-related behaviors of adults in the future, but also correlate to changes in the future disease burden [1,2]. Therefore, national health policy must promote a healthy lifestyle during adolescence. Before the coronavirus disease 2019 (COVID-19) pandemic, the prevalence of current cigarette smoking and current alcohol drinking among Korean adolescents showed decreasing trends, while the prevalence of obesity among adolescents showed an increasing trend [3]. However, the COVID-19 pandemic, which began in 2020, has had a substantial effect on the lifestyle behaviors of adults across the world [4,5] in addition to those of Korean adolescents [6,7].

Beginning in March 2020, in-person educational courses were suspended for over a year due to the COVID-19 pandemic. As a result, social interaction and social support from teachers and other peers have been limited, and adolescents began to spend most of their time at home when learning and in everyday life [8,9]. In addition to affecting adolescents’ educational experiences and social interactions, the COVID-19 pandemic has also influenced the health-related lifestyles of adolescents and adolescent obesity. A recent systematic review reported that the use of substances, including tobacco and alcohol, showed decreasing trends during the COVID-19 pandemic [10]. However, previous studies have also observed a decrease in physical activity [11], and intake of high-calorie snacks and unhealthy foods has increased [12,13] along with the prevalence of obesity [13] in adolescents during the COVID-19 pandemic.

A report from Korea found that consumption of soda, sweet drinks, and fast foods among adolescents decreased in 2020 compared to 2019, with vigorous and moderate physical activity also decreasing during the same period [6]. However, the results of previous studies were somewhat different from the official findings of the Korea Youth Risk Behavior Survey (KYRBS). In addition, few studies have examined changes in lifestyle and the prevalence of obesity among adolescents before and during the COVID-19 pandemic, including major health behaviors such as cigarette smoking and alcohol drinking.

Therefore, this study aimed to comprehensively examine changes in health behaviors and obesity before and during the COVID-19 pandemic using the KYRBS database, which is nationally representative of all Korean students.

## MATERIALS AND METHODS

### Data source

We used KYRBS data from 2015 to 2021 to examine changes in health behaviors and obesity in Korean adolescents before and during the COVID-19 pandemic. The KYRBS database was divided into 2 datasets (2018-2021 and 2015-2021) according to the purpose of the analysis. Pre-pandemic (2018-2019) and pandemic-era (2020-2021) changes in health behaviors and obesity were compared to determine changes in health behaviors and obesity leading up to and during the COVID-19 pandemic. We also used the KYRBS dataset to visually examine the change in the slope of the trend and the differences between data from 2020 and 2021 during the COVID-19 pandemic given previous changes in trends.

The KYRBS is an annual representative cross-sectional survey for all Korean students (middle school to high school) first conducted in 2005 by the Korea Disease Control and Prevention Agency (KDCA) and the Ministry of Education [14]. The 2-step cluster stratified random sampling method is used for the KYRBS to select a representative sample population of Korean students. The first sampling unit refers to the school, and the second sampling unit refers to the class. The KYRBS includes a survey of middle school and high school students by school level for gathering information about health behaviors, mental health, oral health, health equity, and injuries among Korean students. The KYRBS uses a web-based online survey, and response rates tend to be very high, at about 92.9% in the 2021 survey. In addition, the KDCA conducts teacher training programs before the surveys are completed to ensure a standardized data collection process. Standardized procedures are also used to guarantee data quality.

### Health behaviors: cigarette smoking, alcohol drinking and physical activity

The prevalence of current cigarette smoking was defined as the proportion of students who smoked cigarettes for more than 1 day within the previous 30 days [15]. Responses for cigarette smoking were classified into categories that included 0 days (non-smokers), 1-9 days, 10-29 days, and every day to further examine changes in the prevalence of cigarette smoking. The prevalence of current alcohol drinking was defined as the proportion of students who drank at least once within the previous 30 days [16]. Responses for alcohol drinking were classified into categories of 0 days (non-drinkers), 1-5 days, 6-9 days, and ≥ 10 days to further examine changes in the prevalence of alcohol drinking. The prevalence of physical activity was defined as the proportion of students who performed moderately intense physical activity for at least 60 minutes per day for more than 5 days within the previous 7 days regardless of the type of exercise [17]. Responses for physical activity were classified into categories of 0 times/wk, 1-2 times/wk, 3-4 times/wk, and ≥ 5 times/wk to further examine changes in the prevalence of physical activity.

### Dietary behaviors

Fruit consumption was measured based on if the respondents consumed fruits (excluding fruit juice) at least once per day for the previous 7 days [19]. Fast food consumption was measured based on if the respondents ate fast foods, such as pizza, hamburgers, or fried chicken, more than 3 times within the previous 7 days [19].

### Obesity

Body mass index (BMI) was calculated by dividing the weight (kg) by the square of the height (m^2^), which were self-reported in the KYRBS. Obesity in students was defined as those at the 95th percentile or higher based on the age-specific BMI using the 2017 Korean National Growth Charts [18,19].

### Statistical analysis

All analyses were conducted using the “PROC SURVEY” procedure by applying weights given the complex sample design (cluster and strata) by sex. The KYRBS database was divided into 2 datasets (2018-2021 and 2015-2021) according to the purpose of the analysis. At first, we used KYRBS data from 2018 to 2021 to compare behavioral changes before and during the COVID-19 pandemic. The baseline characteristics were presented as numbers and percentages (%). Differences in baseline characteristics and changes in health behaviors, dietary behaviors, and obesity were compared by year range (2018-2019 and 2020-2021) using the chi-square test. In particular, to determine if any changes occurred within each period (pre-pandemic and pandemic), differences between 2018 and 2019 and differences between 2020 and 2021 were compared using the chi-square test. In addition, changes in health behaviors and obesity were examined by sex. The prevalence of health behaviors and obesity in the pre-pandemic period (2018-2019) and during the pandemic (2020-2021) were expressed as percentages (standard error) and examined by sex and school level. Changes in health behaviors and obesity were calculated using absolute differences between the 2 periods. Using complex-sample logistic regression, health behaviors and obesity for all of the participants in all grade level combined and their changes were adjusted for grade level (as a surrogate variable for age).

In addition, we used KYRBS data from 2015 to 2021 to visually examine the change in the slope and the change each year considering previous changes in trends. Linear regression models were used to visually explore the changes in the trend from 2015 to 2019 and the change in the trend from 2020 to 2021 to closely examine the changes each year.

A p-value of < 0.05 was considered statistically significant. All statistical analyses were performed using SAS version 9.4 (SAS Institute Inc., Cary, NC, USA) and Stata version 17.0 (StataCorp., College Station, TX, USA).

### Ethics statement

Ethical approval for the KYRBS was waived by the Enforcement Regulation of Bioethics and Safety Act.

## RESULTS

### Differences in the baseline characteristics of Korean students between the pre-pandemic and pandemic periods

The total number of students who participated in the KYRBS survey during the pre-pandemic and pandemic periods was 117,343 and 109,796, respectively (Table 1). No significant differences were observed in the sex distribution between the pre-pandemic and pandemic periods. However, the proportion of middle school students increased, whereas the proportion of high school students decreased during the pandemic compared to the pre-pandemic period. The prevalence of current cigarette smoking decreased from 6.7% to 4.4% (p<0.001) during the pandemic, and the prevalence of current alcohol drinking also decreased from 16.0% to 10.7% (p<0.001). However, no significant changes in the prevalence of physical activity between the pre-pandemic and pandemic periods were observed (p=0.959). The proportion of students who consumed fast food more than 3 times/wk increased from 23.4% in 2018-2019 to 25.8% in 2020-2021 (p<0.001), whereas the proportion of students who consumed fruit more than once a day decreased significantly from 20.7% to 18.4% (p<0.001). The prevalence of obesity among students increased significantly from 10.9% in 2018-2019 to 12.8% in 2020-2021 (p<0.001). On a year on year basis, no significant differences in the prevalence of obesity between 2018 and 2019 were observed; however, the prevalence of obesity increased significantly by 1.4%p in 2021 compared to 2020 (p<0.001).

### Changes in health behaviors, dietary behaviors, and obesity during the pandemic in male Korean students

**Figure 1.**
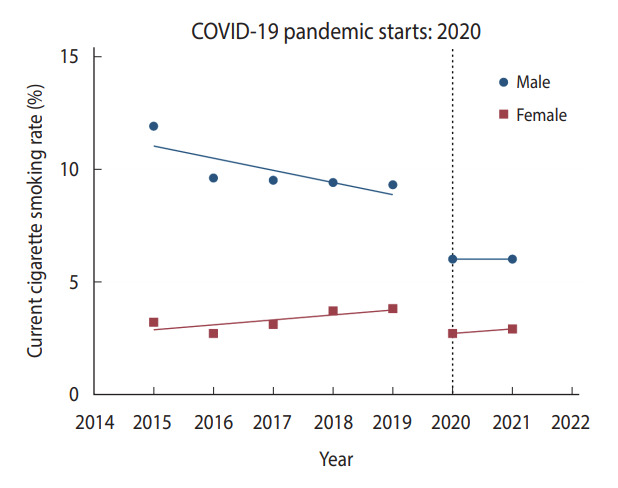
Change in the prevalence of current cigarette smoking among Korean adolescents. Trends in the prevalence of current cigarette smoking from 2015 to 2021 are illustrated. The horizontal dotted line represents the starting point of the coronavirus disease 2019 (COVID-19) pandemic in 2020. The blue dots and straight blue lines represent the observed prevalence of current cigarette smoking and the changing trend in the prevalence of current cigarette smoking for each period in male students, respectively. The red dots and straight red lines represent the observed prevalence of current cigarette smoking and the changing trend in the prevalence of current cigarette smoking for each period in female students, respectively.

**Figure 2.**
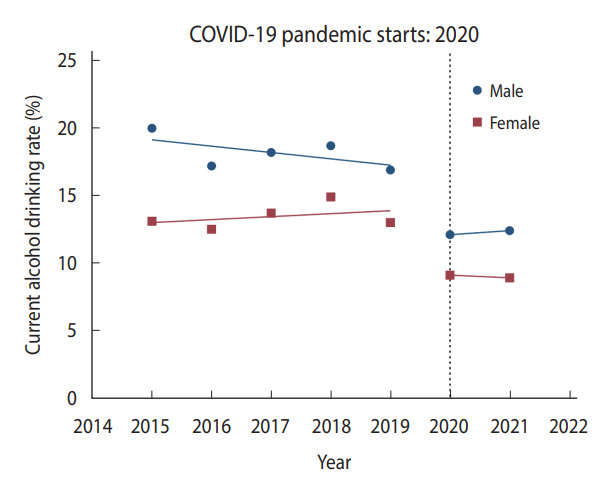
Change in the prevalence of current alcohol drinking among Korean adolescents. Trends in the prevalence of current alcohol drinking from 2015 to 2021 are illustrated. The horizontal dotted line represents the starting point of the coronavirus disease 2019 (COVID-19) pandemic in 2020. The blue dots and straight blue lines represent the observed prevalence of current alcohol drinking and the changing trend in the prevalence of current alcohol drinking for each period in male students, respectively. The red dots and straight red lines represent the observed prevalence of current alcohol drinking and the changing trend in the prevalence of current alcohol drinking for each period in female students, respectively.

**Figure 3.**
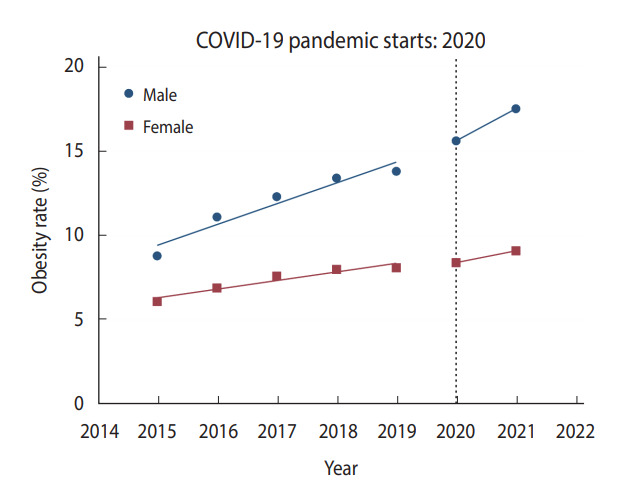
Change in the prevalence of obesity among Korean adolescents. Trends in the prevalence of obesity from 2015 to 2021 are illustrated. The horizontal dotted line represents the starting point of the coronavirus disease 2019 (COVID-19) pandemic in 2020. The blue dots and straight blue lines represent the observed obesity prevalence and the changing trend in the prevalence of obesity for each period in male students, respectively. The red dots and straight red lines represent the observed obesity prevalence and the changing trend in the prevalence of obesity for each period in female students, respectively.

In male Korean students, significant changes in health behaviors and obesity were observed during the pandemic compared to the pre-pandemic period (Table 2). The prevalence of current cigarette smoking, current alcohol drinking, and physical activity among male students significantly decreased during 2020-2021 compared to 2018-2019 (Figures 1 and 2, Supplementary Material 1). Compared to 2020, there was little change in the prevalence of current cigarette smoking in 2021, while the prevalence of physical activity in male students increased slightly (Figure 1, Supplementary Material 1). While the dietary behaviors of male students worsened during 2020-2021 compared to 2018-2019 (Table 2), the increasing trend in male students’ fast food consumption slowed in 2020-2021 compared to the increasing trend in 2015-2019 (Supplementary Material 2). The fruit consumption of male students showed a similar decreasing trend during the COVID-19 pandemic compared to before the pandemic (Supplementary Material 3). The prevalence of obesity among male students increased sharply during the same period (Figure 3). In particular, the prevalence of obesity in male students after the pandemic increased more rapidly compared to before the pandemic.

The prevalence of current cigarette smoking and current alcohol drinking among male high school students decreased more than those of male middle school students. The prevalence of physical activity in male high school students did not show a significant change (p=0.737), whereas the prevalence of physical activity in male middle school students decreased by 1.8%p (p<0.001). The prevalence of obesity among male middle school students increased by 4.1%p during the pandemic, which was much larger than the change in male high school students (2.2%).

### Changes in health behaviors, dietary behaviors, and obesity during the pandemic in female Korean students

Changes in health behaviors, dietary behaviors, and obesity in female students during the pandemic were similar to those in the male students except for the physical activity variable (Table 3). The prevalence of current cigarette smoking and current alcohol drinking among Korean female students significantly decreased in 2020-2021 compared to 2018-2019 (Figures 1 and 2). Interestingly, the prevalence of physical activity among female students increased slightly during the pandemic period (Supplementary Material 1). The dietary behaviors of female students worsened in 2020-2021 compared to 2018-2019 (Table 3, Supplementary Materials 2 and 3). In particular, fruit consumption among female students showed a significant decrease in 2020 compared to 2019 (Supplementary Material 3). The prevalence of obesity among female students increased significantly during the same period (Figure 3).

The prevalence of current cigarette smoking and current alcohol drinking among female high school students decreased more than among female middle school students. The prevalence of physical activity in female high school students did not change significantly (p=0.113), whereas in female middle school students, it increased slightly by 0.8%p (p=0.009). The prevalence of obesity in both female middle school students and high school students similarly increased by 0.7%p and 0.9%p, respectively, during the pandemic.

## DISCUSSION

In this study, changes in health behaviors, dietary behaviors, and obesity among Korean students in 2020-2021 compared to 2018-2019 were examined using KYRBS data. The prevalence of current cigarette smoking and current alcohol drinking decreased during the pandemic compared to the pre-pandemic period, and the prevalence of obesity increased during the same period. In terms of dietary behaviors, fast food consumption increased while fruit consumption decreased during the pandemic. Similar changes were observed between male and female students during the pandemic, except in terms of physical activity.

Changes in the prevalence of obesity and health behaviors in Korean students, including cigarette smoking and alcohol drinking, were similar to changes observed in other countries during the COVID-19 pandemic. In the United States, the National Survey on Drug Use and Health reported that, among United States adolescents aged 12-17 years old, the prevalence of cigarette use decreased from 2.3% in 2019 to 1.4% in 2020 and the prevalence of alcohol use decreased from 9.4% in 2019 to 8.2% in 2020 [20]. A recent systematic review reported that the prevalence of smoking and drinking among adolescents decreased during the COVID-19 pandemic compared to before the pandemic around the world [10].

There have also been many reports of increases in obesity and decreases in walking and physical activity in adolescents during the pandemic. A long-term cohort study of adolescents from the United States aged 2-19 years old who visited the hospital from 2018 to 2020 reported a sharp increase in BMI among adolescents during the COVID-19 pandemic [21]. In addition, 38 pediatric cohorts across the United States reported a sharp increase in BMI in children and adolescents during the pandemic compared to the pre-pandemic period [22]. The increase in obesity among children and adolescents was likely caused by the deterioration of healthy dietary behaviors due to an increase in food insecurity, an increase in unhealthy dietary habits and high-calorie food intake, and a decrease in physical activity due to school closures and lockdowns [23]. In our study, as reported in other countries, adolescents’ dietary behaviors tended to worsen while physical activity did not show significant differences when compared to the pre-pandemic period. This is partially because, unlike other countries, Korea did not implement a strong lockdown policy and mainly relied on mitigation measures such as voluntary social distancing [5,24].

Several other studies have examined changes in the dietary behaviors of adolescents during the COVID-19 pandemic. In the United States, several studies have reported that food insecurity increased during the COVID-19 pandemic, especially in children and adolescents, compared to before the COVID-19 pandemic [25,26]. An increase in the consumption of sweet packaged snacks and processed meats among children was reported during the early lockdown period in 2020 in Italy [27]. In Greece, however, children’s weights were found to increase between April and May 2020, even as children’s consumption of fresh food and fast food increased and decreased, respectively, during the same period [28]. Unlike Korea, many other countries have implemented strong lockdown measures to prevent the spread of COVID-19 [24]; thus, we can infer that food accessibility and food security in Korea differed from other countries. Indeed, in Japan, which faced a similar situation to Korea during the COVID-19 pandemic, well-balanced dietary behaviors were found to have significantly decreased after a state of emergency was declared regarding COVID-19 [29].

One advantage of this study is that our findings are representative of Korean students overall due to our use of a large-scale nationwide survey, the KYRBS. Second, changes between 2020 and 2021 were compared, and changes were examined in further detail by conducting analyses based on sex and school level. Nevertheless, there are several limitations to be aware of when interpreting our study findings. First, health behaviors and obesity (BMI) may have been underestimated or overestimated since the questionnaires to assess students’ health behaviors depended on self-reported answers. In general, when data are self-reported, reported heights and weights tend to be higher and lower, respectively [30]. In addition, although the KYRBS is representative of all Korean students, it does not include adolescents who are not currently attending school. Therefore, our study findings do not necessarily represent all adolescents in Korea. Additionally, some variables may have been affected by seasonal variation. The KYRBS was conducted from June to August before the COVID-19 pandemic (in 2018 and 2019), whereas after the COVID-19 pandemic, the survey was conducted from August to November. This difference in the survey period may have affected some survey items that could be affected by seasonality, such as physical activity or fruit consumption. Another limitation of this study is that the use of e-cigarettes and heated tobacco products was not included. Only cigarettes were included to determine the smoking prevalence. The proportion of e-cigarette and heated tobacco product users among adolescents is higher than among adults. Finally, it would be necessary to examine the trends after the end of the COVID-19 pandemic to confirm whether these changes were actually caused by the pandemic.

## CONCLUSION

In conclusion, the prevalence of obesity among Korean adolescents increased while the prevalence of cigarette smoking and alcohol drinking decreased during the pandemic. In addition, Korean adolescents’ dietary behaviors worsened during the same period. Since the deterioration of adolescent dietary behaviors and an increase in the prevalence of obesity could increase the future disease burden, concerted efforts are needed at the individual and national levels to reduce obesity and promote healthy dietary behaviors in Korean adolescents.

## Figures and Tables

**Figure f4-epih-45-e2023018:**
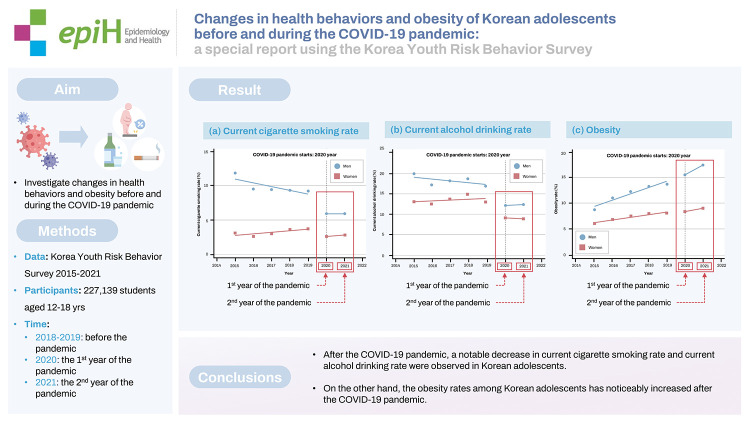


**Table 1.** Baseline characteristics of the study participants

Characteristics		Pre-pandemic		p-value^1^	Pandemic		p-value^2^	Pre-pandemic	Pandemic	p-value^3^
		2018	2019		2020	2021		2018-2019	2020-2021	
Total		60,040 (100)	57,303 (100)		54,948 (100)	54,848 (100)		117,343 (100)	109,796 (100)	
Sex										0.843
	Male	30,463 (52.1)	29,841 (52.0)	0.959	28,353 (51.9)	28,401 (51.7)	0.912	60,304 (52.0)	56,754 (51.8)	
	Female	29,577 (47.9)	27,462 (48.0)		26,595 (48.1)	26,447 (48.3)		57,039 (48.0)	53,042 (48.2)	
School level										<0.001
	Middle school	30,229 (46.4)	29,384 (47.9)	0.215	28,961 (49.6)	30,015 (51.0)	0.229	59,613 (47.1)	58,976 (50.3)	
	High school	29,811 (53.6)	27,919 (52.1)		25,987 (50.4)	24,833 (49.0)		57,730 (52.9)	50,820 (49.7)	
Region										0.434
	City (si)	26,654 (42.8)	25,335 (42.5)	0.768	23,621 (42.2)	23,862 (41.9)	0.778	51,989 (42.7)	47,483 (42.1)	
	Province (do)	33,386 (57.2)	31,968 (57.5)		31,327 (57.8)	30,986 (58.1)		65,354 (57.3)	62,313 (57.9)	
Cigarette smoking										<0.001
	Smoker	3,722 (6.7)	3,693 (6.7)	0.961	2,470 (4.4)	2,404 (4.5)	0.757	7,415 (6.7)	4,874 (4.4)	
	Non-smoker	56,318 (93.3)	53,610 (93.3)		52,478 (95.6)	52,444 (95.5)		109,928 (93.3)	104,922 (95.6)	
Alcohol drinking										<0.001
	Drinker	9,667 (16.9)	8,400 (15.0)	<0.001	5,892 (10.7)	5,803 (10.7)	0.824	18,067 (16.0)	11,695 (10.7)	
	Non-drinker	50,373 (83.1)	48,903 (85.0)		49,056 (89.3)	49,045 (89.3)		99,276 (84.0)	98,101 (89.3)	
Physical activity										0.959
	Physically active^4^	8,585 (13.9)	8,782 (14.7)	0.032	8,131 (14.0)	8,351 (14.6)	0.105	17,367 (14.3)	16,482 (14.3)	
	Physically inactive	51,455 (86.1)	48,521 (85.3)		46,817 (86.0)	46,497 (85.4)		99,976 (85.7)	93,314 (85.7)	
Fast food consumption (times/wk)										<0.001
	≥3	12,585 (21.4)	14,392 (25.5)	<0.001	13,656 (25.4)	14,245 (26.2)	0.021	26,977 (23.4)	27,901 (25.8)	
	<3	47,455 (78.6)	42,911 (74.5)		41,292 (74.6)	40,603 (73.8)		90,366 (76. 6)	81,895 (74.2)	
Fruit consumption (times/day)										<0.001
	≥1	12,366 (20.8)	11,485 (20.5)	0.281	10,001 (18.7)	9,786 (18.1)	0.082	23,851 (20.7)	19,787 (18.4)	
	<1	47,674 (79.2)	45,818 (79.5)		44,947 (81.3)	45,062 (81.9)		93,492 (79.3)	90,009 (81.6)	
Obesity										
	Obese^5^	6,335 (10.8)	6,305 (11.1)	0.316	6,679 (12.1)	7,328 (13.5)	<0.001	12,640 (10.9)	14,007 (12.8)	<0.001
	Non-obese	52,001 (89.2)	49,443 (88.9)		46,855 (87.9)	46,117 (86.5)		101,444 (89.1)	92,972 (87.2)	

Values are presented as number (%). The Rao-Scott chi-square test was used considering the complex survey design to compare the baseline characteristics across the categorical variables.

1 The difference in the distribution of variables between 2018 and 2019 was examined using the Rao-Scott chi-square test.

2 The difference in the distribution of variables between 2020 and 2021 was examined using the Rao-Scott chi-square test.

3 The difference in the distribution of variables between the pre-pandemic period and the pandemic was examined using the Rao-Scott chi-square test.

4 Physically active adolescents were defined as the proportion of students who participated in moderately intense physical activity for at least 60 min/day for more than 5 days within the previous 7 days regardless of the type of exercise.

5 Obese adolescents were defined as those at the 95th percentile or higher based on the age-specific body mass index for each age group using the 2017 Korean National Growth Charts.

**Table 2.** Changes in the prevalence of health behaviors and obesity after the COVID-19 pandemic among male Korean adolescents

Variables			Pre-pandemic	Pandemic	Changes^1^	p-value^2^
			2018-2019	2020-2021	%p	
Current cigarette smoking						
	Total^3^		9.4 (0.2)	6.0 (0.2)	-3.4	<0.001
	School level					
		Middle school	3.9 (0.1)	2.0 (0.1)	-1.9	<0.001
		High school	14.2 (0.3)	10.0 (0.3)	-4.2	<0.001
Current alcohol drinking						
	Total^3^		17.8 (0.3)	12.3 (0.2)	-5.5	<0.001
	School level					
		Middle school	8.7 (0.2)	6.0 (0.2)	-2.7	<0.001
		High school	25.8 (0.4)	18.6 (0.3)	-7.2	<0.001
Physical activity^4^						
	Total^3^		20.9 (0.2)	20.3 (0.2)	-0.6	0.007
	School level					
		Middle school	24.6 (0.3)	22.8 (0.3)	-1.8	<0.001
		High school	17.5 (0.3)	17.7 (0.3)	0.2	0.737
Fast food consumption (≥3 times/wk)						
	Total^3^		25.0 (0.2)	27.6 (0.2)	2.6	<0.001
	School level					
		Middle school	23.3 (0.3)	25.5 (0.3)	2.2	<0.001
		High school	26.5 (0.3)	29.7 (0.3)	3.2	<0.001
Fruit consumption (≥1 time/day)						
	Total^3^		20.6 (0.2)	18.8 (0.2)	-1.8	<0.001
	School level					
		Middle school	24.3 (0.3)	22.0 (0.3)	-2.3	<0.001
		High school	17.2 (0.3)	15.6 (0.3)	-1.6	<0.001
Obesity^5^						
	Total^3^		13.6 (0.2)	16.6 (0.2)	3.0	<0.001
	School level					
		Middle school	11.9 (0.2)	16.0 (0.2)	4.1	<0.001
		High school	15.0 (0.2)	17.2 (0.3)	2.2	<0.001

Values are presented as % (standard error).

1 The changes in prevalence rates were calculated as the absolute difference in the prevalence rates between 2018-2019 and 2020-2021.

2 Logistic regression analysis was used considering the complex survey design to compare the differences across the categorical variables.

3 The health behaviors and obesity of the total participants in all grade level combined and their changes were adjusted for the grade level.

4 Physically active adolescents were defined as the proportion of students who participated in moderately intense physical activity for at least 60 min/day for more than 5 days within the previous 7 days regardless of the type of exercise.

5 Obese adolescents were defined as those at the 95th percentile or higher based on the age-specific body mass index for each age group using the 2017 Korean National Growth Charts.

**Table 3.** Changes in the prevalence of health behaviors and obesity after the COVID-19 pandemic among female Korean adolescents

Variables			Pre-pandemic	Pandemic	Changes^1^	p-value^2^
			2018-2019	2020-2021	%p	
Current cigarette smoking						
	Total^3^		3.8 (0.1)	2.8 (0.1)	-1.0	<0.001
	School level					
		Middle school	2.2 (0.1)	1.6 (0.1)	-0.6	<0.001
		High school	5.2 (0.2)	4.0 (0.2)	-1.2	<0.001
Current alcohol drinking						
	Total^3^		14.0 (0.3)	9.0 (0.2)	-5.0	<0.001
	School level					
		Middle school	7.2 (0.2)	5.0 (0.2)	-2.2	<0.001
		High school	20.1 (0.4)	13.1 (0.3)	-7.0	<0.001
Physical activity^4^						
	Total^3^		7.2 (0.1)	7.9 (0.1)	0.7	0.003
	School level					
		Middle school	9.0 (0.2)	9.8 (0.2)	0.8	0.009
		High school	5.5 (0.2)	6.0 (0.2)	0.5	0.113
Fast food consumption (≥3 times/wk)						
	Total^3^		21.7 (0.2)	23.9 (0.2)	2.2	<0.001
	School level					
		Middle school	20.7 (0.3)	21.9 (0.3)	1.2	<0.001
		High school	22.6 (0.3)	25.8 (0.3)	3.2	<0.001
Fruit consumption (≥1 time/day)						
	Total^3^		20.8 (0.2)	17.9 (0.2)	-2.9	<0.001
	School level					
		Middle school	24.4 (0.3)	20.8 (0.8)	-3.6	<0.001
		High school	17.5 (0.3)	15.1 (0.3)	-1.6	<0.001
Obesity^5^						
	Total^3^		8.1 (0.1)	8.8 (0.1)	0.7	<0.001
	School level					
		Middle school	6.1 (0.2)	6.8 (0.2)	0.7	0.004
		High school	9.9 (0.2)	10.8 (0.2)	0.9	0.002

Values are presented as % (standard error).

1 The changes in prevalence rates were calculated as the absolute difference in the prevalence rates between 2018-2019 and 2020-2021.

2 Logistic regression analysis was used considering the complex survey design to compare the differences across the categorical variables.

3 The health behaviors and obesity of the total participants in all grade level combined and their changes were adjusted for the grade level.

4 Physically active adolescents were defined as the proportion of students who participated in moderately intense physical activity for at least 60 minutes per day for more than 5 days within the previous 7 days regardless of the type of exercise.

5 Obese adolescents were defined as those at the 95th percentile or higher based on the age-specific body mass index for each age group using the 2017 Korean National Growth Charts.

## SUPPLEMENTARY MATERIALS

Supplementary Material 1. Baseline characteristics of the study participants

Supplementary Material 2 Changes in the prevalence of health behaviors and obesity after the COVID-19 pandemic among male Korean adolescents

Supplementary Material 3 Changes in the prevalence of health behaviors and obesity after the COVID-19 pandemic among female Korean adolescents
